# Is Optical Coherence Tomography a Useful Tool to Objectively Detect Actual Posterior Vitreous Adhesion Status?

**DOI:** 10.1155/2016/3953147

**Published:** 2016-02-16

**Authors:** Thomas Bertelmann, Christina Goos, Walter Sekundo, Stephan Schulze, Stefan Mennel

**Affiliations:** ^1^Department of Ophthalmology, Philipps-University Marburg, 35037 Marburg, Germany; ^2^Department of Ophthalmology, Feldkirch State Hospital, 6800 Feldkirch, Austria

## Abstract

*Purpose*. To objectively detect true posterior vitreous cortex (PVC) adhesion status using a commercially available swept-source OCT device (DRI OCT-1, Atlantis^©^).* Material and Methods*. Case report, review of the literature, and methodical discussion of concepts to improve OCT-guided PVC imaging.* Results*. Standard OCT imaging misdiagnosed PVC adhesion status as totally detached in this case report when using a horizontal 6 mm scan only. Contrariwise imaging the same eye with a 12 mm horizontal scan, partial posterior vitreous detachment (PVD) and the presence of a bursa premacularis were clearly discernible. Besides a broader scan, specific scan patterns, highest resolution, and contrast sensitivity, an anterior-to-posterior adjusted scan through the entire vitreous as well as the detection of characteristic undulating aftermovements might enhance the capability of OCT imaging to detect true PVC adhesion status.* Conclusions*. Further developments are needed to address these issues and to establish OCT recordings as the standard and objective method of choice in PVC adhesion status imaging.

## 1. Introduction

The interrelations between the posterior vitreous cortex (PVC) and the internal limiting membrane (ILM) of the neurosensory retina and the impact in regard to health and disease of the vitreoretinal interface (VRI) advanced into the focus of vitreoretinal research within the last decades [1]. An attached or partially detached PVC, also known as anomalous posterior vitreous detachment (PVD) [2], is attributed to play a significant role in the development and progression of various vitreoretinal diseases whereas a complete PVD might serve as a preventive element [1, 3–7]. Prior to the OCT era the evaluation of PVC adhesion status was limited to B-scan ultrasound and indirect biomicroscopy, both of which are techniques which strongly rely on the examining physicians' experience [1]. To date OCT analysis is considered as the gold standard of VRI imaging [8] and conditions like vitreomacular adhesion (VMA) and vitreomacular traction (VMT) are clearly discernible [9]. Contrariwise, if the PVC remains completely attached or is totally detached and shifted far anteriorly out of the scanning frame, OCT imaging oftentimes fails to detect the accurate PVC adhesion status [1]. This aspect might be accountable for the observation that overall OCT diagnosis matches only in 12,5% with true PVC adhesion status as determined during triamcinolone-assisted pars plana vitrectomy [10]. The case presented herein demonstrates one example where accurate determination of PVC adhesion status was challenging. It further summarizes current and potential prospective technical features and options to enhance PVC visibility in all eyes.

## 2. Material and Methods

This report is on a 50-year-old male patient complaining about a representative vitreous floater pathology. Standard OCT scans of the macula and VRI of both eyes were performed using the DRI OCT-1, Atlantis^©^ swept-source OCT (Topcon Medical Systems, Oakland, NJ). The patient was seated in front of the OCT device in an upright position and asked to focus on the fixating light. A moderate myopic refraction was adjusted before imaging. One horizontal 12 mm scan was conducted including the optic nerve head (ONH) as well as the fovea centralis. By default, an A-scan rate of 100000 Hz was used. The incorporated light source is a wavelength-tunable laser, centered at 1050 nm with a 100 nm tuning range. Thus an axial resolution of 8 *μ*m, a lateral resolution of 20 *μ*m, and an imaging depth of 2.3 mm can be obtained [11]. Patient gave his consent to publishing of this case report.

## 3. Results

**Figure 1:**
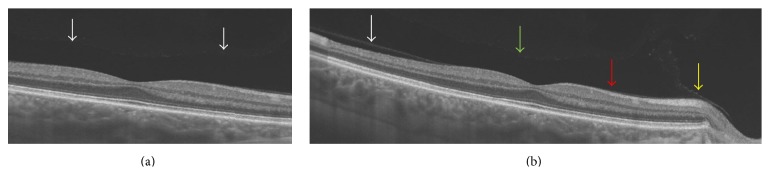
(a) Central horizontal 6 mm scan: the PVC seems to be completely detached (total PVD) (white arrows). (b) Complete horizontal 12 mm scan: partial PVD including an attached PVC centrally heading towards the ONH (red arrow) and a shallow PVD laterally (white arrow); the preretinal hyporeflective structure (between retina and green arrow) displays the posterior precortical vitreous pocket (PPVP), also called bursa premacularis. The green arrow depicts the anterior boundary of the PPVP; the yellow arrow focusing the septum interpapillomaculare.

The results of his right eye are illustrated in Figure 1. Overall, no vitreoretinal pathology was discernible. Examination of the 6 mm macular scan in Figure 1(a) omits the optic nerve, fails to detect any posterior vitreous structures, and is suggestive of complete posterior vitreous separation, consistent with the patients presenting complaint. Cursory examination of the wider 12 mm scan in Figure 1(b) may suggest at least a partial posterior vitreous separation in the temporal perifovea (white arrow). However, the overlying optically empty structure corresponding to the premacular bursa (green arrow) and the fine layer of cortex (red arrow) as well as the septum interpapillomaculare (yellow arrow) originating from the temporal aspect of the optic nerve establish unequivocally that the temporal structure is indeed the posterior wall of the premacular bursa and that the vitreous is in fact completely attached.

## 4. Discussion

**Figure 2:**
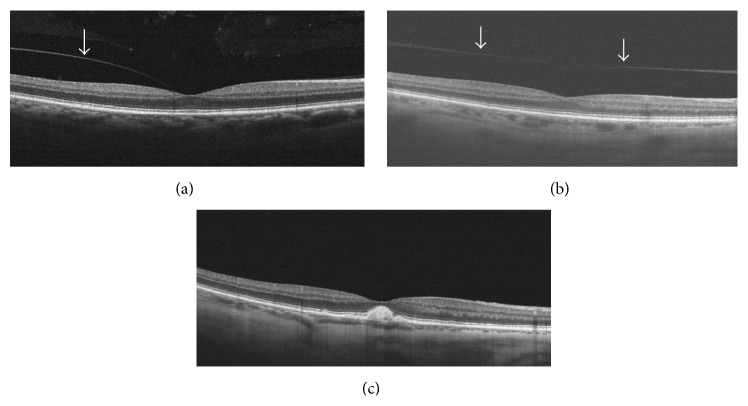
(a) VMA and partial PVD; the PVC is clearly discernible (white arrow). (b) Complete but shallow PVD: the completely detached PVC is definitely presentable, because PVC is shifted only little anteriorly within the scanning frame (white arrow). (c) In this case, PVC adhesion status cannot be identified, because PVC is not visible (i.e., completely attached or totally detached).

The identification of true PVC adhesion status frequently turns out to be challenging. Nevertheless, in the era of commercially available proteases (Jetrea^©^, Alcon Pharma GmbH, Freiburg im Breisgau, Germany) to induce PVD development within the scope of the so-called enzymatic vitreolysis [12] the detection of true PVC adhesion status is essential to treat patients with persistent vitreoretinal attachment and simultaneously to avoid intravitreal injections in eyes with already existing total PVD. In contrast to B-scan ultrasound and biomicroscopy, both of which are in the end at least in part subjective assessments of the VRI, imaging of these structures with a standardized and objective measurement tool like OCT is of clear benefit [1]. As demonstrated in Figure 2(a) standardized OCT imaging can definitely define PVC adhesion status in cases of VMA and VMT, both of which are subsumed as partial or anomalous PVD [2], as well total PVD, while the completely detached PVC remains closely anterior to the retina within the scanning frame (Figure 2(b)), which in turn is oftentimes not the case. So far OCT imaging is unable to discriminate between a completely attached and totally detached PVC that has shifted anteriorly out of the scanning frame as depicted in Figure 2(c) though.

If there was an OCT tool to overcome this particular problem, true PVC adhesion status might be analyzed in all eyes, but so far, such a VRI tool is not available. This in turn might increase the objective identification of correct PVC adhesion status in all eyes which was recently calculated to be only 12.5% [10] and thus justify the indication whether or not to intravitreally inject ocriplasmin (Jetrea^©^) to induce PVD development.

Meanwhile there are some approaches to address this issue. First, the length of a single OCT scan centered in the fovea is of particular importance as demonstrated in this case report. While some OCT devices measure 6 mm by default, others analyze 12 mm [13–16]. As demonstrated herein, if only 6 mm is depicted PVC adhesion status might be misinterpreted. A broader scan including the ONH as well as the temporal aspect of the macula will definitely help to detect the objective anatomic interrelations within the VRI [17]. Hence some of the OCT devices can be upgraded or will soon have the option to upgrade with wide field lenses to increase VRI diagnostic abilities.

Second, the scan pattern applied can help to enhance the visibility of the different structures within the VRI. While a single scan centered in the fovea might miss important details, the use of radial or raster scans of the macula can unfold otherwise missed details [17]. Furthermore, radial scans might be superior in comparison to raster scans to detect true PVC adhesion status [18].

Third, the highest resolution and optimal contrast sensitivity of images obtained can help to visualize the different structures in the VRI. In regard to the highest resolution no profound differences exist between the up-to-date and commercially available SD-OCT devices (5 *μ*m to 10 *μ*m) [13–16]. Nevertheless future OCT generations will increase the highest resolution and thus enhance the visibility of intraocular eye structures [19]. Basically, single B-scans have a higher resolution than imaging in a 3-dimensional acquisition mode and thus should be used for best quality results [20]. In regard to contrast issues, optimizing the contrast sensitivity manually might be a better option than using automatic contrast function [14]. Future developments in respect to both variables will potentially help to distinguish between the PVC and the ILM of the neurosensory retina even if the PVC is still completely attached. This aspect is of special interest, because both structures might have equal optical properties and the principal of OCT technique is to detect differences between two optical unequal structures like the fundamentals of ultrasound. If a differentiated presentability of both structures turns out to be technically possible in the future, that might be the crucial step in objective VRI imaging.

Fourth, if this discrimination remains an insolvable issue in the future, another possibility of detecting true PVC adhesion status might be an anterior-to-posterior adjusted scan through the entire vitreous [21, 22], because even if the PVC is completely detached and shifted anteriorly out of the standard image frame of 1.6 mm to 2.3 mm anteriorly to the ILM [13–16], then it still should be clearly discernible. So far there is no device available with such an anterior-to-posterior adjusted scan of the entire vitreous, but swept-source OCT technology might be an option to realize this consideration.

Fifth, there are attempts to visualize the “invisible” vitreous and the PVC by shifting the focus into the posterior vitreous cavity by adding +2 dpt or +4 dpt during routine OCT examinations [17], which in turn advances the visibility of preretinal structures like PPVP [14, 17]. The limitation remains that the scanning frame persists between 1.6 and 2.3 mm at most though. Even if the retina is shifted posteriorly on the screen while performing OCT imaging and the head of the OCT device is moved slightly anteriorly and thus away from the patients' eyes, a significant enlargement of the frame is not attainable [11, 13, 23]. Here, the problem arises that the retina is needed as a reference structure. Without the latter almost no image can be obtained.

Sixth, when using B-scan ultrasound to analyze PVC adhesion status, characteristic undulating aftermovements (motion of the hyaloid observed on the screen after cessation of eye movement) oftentimes help to determine the posterior hyaloid as detached. So far it is hardly possible to perform OCT imaging in a moving eye. This might be another possibility of enhancing OCT-guided VRI predictability.

Seventh, further routine and experimental techniques are available to enhance the visibility of the structures in the VRI, including the use of an eye tracker in combination with image-averaging software to obtain multiple images from the same exact location and thus to reduce the signal-to-noise-ratio (e.g., Heidelberg Spectralis HRA; Heidelberg Engineering, Carlsbad, California, USA). In an experimental setting a combination of SLO and OCT to track the posterior vitreous hyaloid was successfully performed [20]. There are further reports on new technologies like enhanced vitreous imaging (EVI) or combined depth imaging (CDI) to improve the visibility of VRI structures [16, 17].

Worst described the bursa premacularis as “a well-defined fluid-filled space inside the vitreous body in front of the macula” [24]. Kishi and Shimizu were able to demonstrate the PPVP in human autopsy eyes in a biomicroscopic evaluation. Here, the posterior wall was “composed of a thin layer of vitreous cortex. The anterior border was contoured by the formed vitreous” [25]. Further publications reported on PPVP development from early childhood on [26, 27]. Finally, Sebag recently discussed exceeding gel liquefaction over vitreoretinal dehiscence as a “unifying concept” for anomalous PVD and coexistent PPVP development [2].

## 5. Conclusion

All these attempts have advanced the ability to distinguish the different anatomical characteristics in the VRI like the PPVP/bursa premacularis, the area of Martegiani, Cloquet's canal, and Eisner's hyaloid tract. Nevertheless, the differentiation of a completely attached and totally detached PVC remains challenging if not impossible up to date with recent OCT devices. Consequently, a combination of OCT and B-scan ultrasound imaging is indicated so far for appropriate PVC adhesion determination [28]. Further developments are needed to address these issues discussed herein and to establish OCT recordings as the standard and objective method of choice in PVC adhesion status imaging.
